# Have Non-physician Clinicians Come to Stay? Comment on "Non-physician Clinicians in Sub-Saharan Africa and the Evolving Role of Physicians"

**DOI:** 10.15171/ijhpm.2016.86

**Published:** 2016-06-29

**Authors:** Gottlieb Lobe Monekosso

**Affiliations:** ^1^Regional Office for Africa, World Health Organization (WHO), Republic of Congo, Africa.; ^2^Global Health Dialogue, Buea, Cameroon.

**Keywords:** Non-physician Clinician (NPC), Physician, Tradi-Practitioner, Health Worker, Healthcare Workforce, Medical Education

## Abstract

A decade ago, sub-Saharan Africa accounted for 24% of the global disease burden but was served by only 4% of the global health workforce. The chronic shortage of medical doctors has led other health professionals especially nurses to perform the role of healthcare providers. These health workers have been variously named clinical officers, health officers, physician assistants, nurse practitioners, physician associates and non-physician clinicians (NPCs) defined as "health workers who have fewer clinical skills than physicians but more than nurses." Although born out of exigencies, NPCs, like previous initiatives, seem to have come to stay and many more medical doctors are being trained to care for the sick and to supervise other health team members. Physicians also have to assume new roles in the healthcare system with consequent changes in medical education

Eyal et al stated that a decade ago, sub-Saharan Africa accounted for 24% of the global disease burden but was served by only 4% of the global health workforce.^1^ Currently, this region has a severe deficit of health professionals. This shortage has led to task shifting and the “creation” of new categories described as non-physician clinicians (NPCs) now active in the majority of countries.^1^ The terminology NPC suggests to us that the mother prototype is the physician to whom societal members agree to confer the solution of their health problems. The physician shares this responsibility with other health professionals. NPC also suggests substitute physicians playing a predominantly subsidiary patient - care role in their home country. Many NPCs aspire to become physicians.

Has there ever been an adequate number of physicians (medical doctors) in sub-Saharan Africa? This is why over the decades other health professionals especially nurses have assumed the role of healthcare providers.^2^ Medicine and healthcare have evolved and continue to evolve with changes in society.^2^ During the colonial period, only a small number of qualified European physicians were available. Overwhelmed by the workload, they delegated professional responsibilities to African nurses and technicians. These, we believe, were the first NPCs. Recognizing that this was a permanent situation, they undertook training of the African assistants informally at first and later in small training centres. The need for NPCs was felt especially in emergency situations like epidemics, disasters and in wartime. They were variously called clinical officers, health officers, physician assistants, nurse practitioners, nurse clinicians, and physician associates. NPCs have been defined as “health workers who have fewer clinical skills than physicians but more than nurses.” It has been noted that in spite of their shorter training they were capable of many diagnostic and therapeutic tasks of physicians. The value for NPCs has been felt in circumstances calling for increased demand for healthcare such as happened in the pandemic of tuberculosis and its mass treatment in the mid twentieth century and now in the HIV/AIDS pandemic in the current century.

One would also ask, does the emergence of a new category of health workers justify a new title of “NPC health system” even if they are performing a significant portion of diagnostic and therapeutic tasks traditionally performed by physicians?^1^ This would look like reconstructing the health system each time a new category is introduced. Rather, one would speak of members of health teams headed by physicians and integrating NPCs, nurses, and midwives, laboratory and sanitary technicians, etc as in other parts of the world. We should also note that in spite of acceptance of the services of NPCs in many African countries, these same countries are scaling up the numbers of medical doctors trained in rapidly increasing numbers of medical schools. It would appear that the more NPCs are trained, the more medical doctors are needed.^1^

## Physicians’ Role in Healthcare Delivery in Sub-Saharan Africa

This is a longstanding debate. It has long been recognized that physicians working in Africa have an expanded role compared to their counterparts working in Europe.^3^ Indeed, European doctors working in Africa during the colonial era splendidly undertook additional functions in spite of what might have been considered short falls in their training, which was, in some instances “completed” by courses in tropical medicine. The pioneer medical training institutions (Dakar, Lagos, Leopoldville, Kampala, Madagascar) recognized the need for expanded roles of the physician. The Association of Medical Schools in Africa (AMSA) and the World Health Organization (WHO) confirmed these ideas which were concretized in 1969 at the University Centre for Health Sciences created in Yaounde and revisited at the World Conference of Medical Education in Edinburgh in 1988.^4^ This international multi-country WHO/UNDP supported project was charged with implementing the then new ideas recalled in the table presented in the editorial entitled “*Potential New Competency Domains and a Model of Associated New Tasks for Physicians in NPC Based Health Systems.*” Those who have experienced developments in sub-Saharan Africa will recognize in the model associated new tasks for physicians in existing district health systems. Medical assistants trained in the colonial era medical schools (mentioned above) progressively developed their skills, were promoted to posts of responsibility in district hospitals and public health services to the extent that they became indistinguishable from European trained medical doctors.^5^

## Training Physicians for Their New Roles

Human resources for health (HRH) experts would all agree that training programmes in sub-Saharan Africa should formally and fully prepare physicians for clinical and non-clinical tasks. Many schools and faculties of medicine emphasise biomedical, clinical, and epidemiological sciences but only a few include in the curricula training modules^6,7^ for ethical conduct and building capacity for leadership and team management. Very few can claim pertinent pedagogical areas that foster synergy and cooperation between members of multidisciplinary teams. The Yaounde School negotiated the profile of the doctor in Cameroon with experts of the Ministry of Public Health and came out with a curriculum which has stood the test of time by describing the profile of the expected graduate in behavioural terms. A core curriculum in the first year was shared by other categories of health professionals, followed in all cases by skills and knowledge appropriate to the different health professions.^8^

One of the lessons we should learn from task shifting and the training of “new health professionals” is the difference in skills of the NPCs who have less skills than the physician but more skills than the nurse. The strength of the fully trained professional is related to the depth of teaching the fundamental biomedical and biosocial disciplines. This is what makes them capable of critical scientific thinking, problem-solving, and continuing professional education through self-directed learning.^9^ It is a fact that NPCs’ short training in the fundamental sciences results in the need for repeated seminars and workshops for updates which are often unavailable. Fully trained professionals (doctors and nurses) are more easily able to assume the roles of leadership, mentorship, and technical management.

## Changes in Medical Education

Medical schools are increasingly aware of the need for training physicians for their new roles not only like the ones proposed in the attractive table in the editorial by Eyal et al.^1^ They are targeting relevance and excellence with a view to quality medical education that would ensure quality healthcare. The curriculum would emphasise the relevance to the health needs of local communities but not forgetting excellence appreciated by the international community. There has been a tendency to move away from the teaching of individual subjects separately which would make a total of 40 or more subjects and disciplines. These are now being grouped in related disciplines such as biomedical sciences, clinical sciences and public health sciences to facilitate the adaptation curriculum to local circumstances.

These approaches also open the way to the definition of curricular objectives in behavioural terms such as desirable professional attitudes, critical scientific thinking, carefully selected professional skills alongside a package of professional knowledge. In this context, defining the profiles of the physician would stimulate similar action on the profiles of other health professionals, if this has not already been done, and opening the way for inter-professional education. Future health professionals would learn together so as to be able to work comfortably together for improved health outcomes.

Teachers should be aware that medical students and other health professional trainees spend a better part of a decade preparing for practice and this ought to be an opportunity to bond the future doctor with population groups, health services, research projects, in-country academic and economic opportunities in medical practice, and in this way fight external migration or brain drain. Medical schools should miss this opportunity.

## Ethical issues

Not applicable.

## Competing interests

Author declares that he has no competing interests.

## Author’s contribution

GLM is the single author of the paper.
